# Purinergic Antagonist Suramin Aggravates Myocarditis and Increases Mortality by Enhancing Parasitism, Inflammation, and Reactive Tissue Damage in *Trypanosoma cruzi*-Infected Mice

**DOI:** 10.1155/2018/7385639

**Published:** 2018-09-30

**Authors:** Rômulo D. Novaes, Eliziária C. Santos, Marli C. Cupertino, Daniel S. S. Bastos, Andréa A. S. Mendonça, Eduardo de Almeida Marques-da-Silva, Sílvia A. Cardoso, Juliana L. R. Fietto, Leandro L. Oliveira

**Affiliations:** ^1^Institute of Biomedical Sciences, Department of Structural Biology, Federal University of Alfenas, MG, Brazil; ^2^School of Medicine, Federal University of Jequitinhonha and Mucuri Valleys, MG, Brazil; ^3^Departament of General Biology, Federal University of Viçosa, MG, Brazil; ^4^Department of Medicine and Nursing, Federal University of Viçosa, MG, Brazil; ^5^Department of Biochemistry and Molecular Biology, Federal University of Viçosa, MG, Brazil

## Abstract

Suramin (Sur) acts as an *ecto*-NTPDase inhibitor in *Trypanosoma cruzi* and a P2-purinoceptor antagonist in mammalian cells. Although the potent antitrypanosomal effect of Sur has been shown *in vitro*, limited evidence *in vivo* suggests that this drug can be dangerous to *T. cruzi*-infected hosts. Therefore, we investigated the dose-dependent effect of Sur-based chemotherapy in a murine model of Chagas disease. Seventy uninfected and *T. cruzi*-infected male C57BL/6 mice were randomized into five groups: SAL = uninfected; INF = infected; SR5, SR10, and SR20 = infected treated with 5, 10, or 20 mg/kg Sur. In addition to its effect on blood and heart parasitism, the impact of Sur-based chemotherapy on leucocytes myocardial infiltration, cytokine levels, antioxidant defenses, reactive tissue damage, and mortality was analyzed. Our results indicated that animals treated with 10 and 20 mg/kg Sur were disproportionally susceptible to *T. cruzi*, exhibiting increased parasitemia and cardiac parasitism (amastigote nests and parasite load (*T. cruzi* DNA)), intense protein, lipid and DNA oxidation, marked myocarditis, and mortality. Animals treated with Sur also exhibited reduced levels of nonprotein antioxidants. However, the upregulation of catalase, superoxide dismutase, and glutathione-S-transferase was insufficient to counteract reactive tissue damage and pathological myocardial remodeling. It is still poorly understood whether Sur exerts a negative impact on the purinergic signaling of *T. cruzi*-infected host cells. However, our findings clearly demonstrated that through enhanced parasitism, inflammation, and reactive tissue damage, Sur-based chemotherapy contributes to aggravating myocarditis and increasing mortality rates in *T. cruzi*-infected mice, contradicting the supposed relevance attributed to this drug for the treatment of Chagas disease.

## 1. Introduction

American trypanosomiasis or Chagas disease (ChD) is a neglected tropical illness caused by the protozoan parasite *T. cruzi*, which is the main infectious agent responsible for nonischemic cardiomyopathy worldwide [1, 2]. Approximately 7 million people are infected in Latin America and the Caribbean, and at least 300,000 new cases are diagnosed each year [2, 3]. This disease has high morbidity and is responsible for 14,000 deaths/year from heart failure in South America [3]. Chronic Chagas cardiomyopathy, the most severe manifestation of *T. cruzi* infection, develops in about 30% of infected individuals [4] and accounts as the third indication for heart transplantation in South America [5].

After almost six decades, the chemotherapeutic treatment for ChD continues to be based on nitrocompounds such as benznidazole (Bz) and nifurtimox (discontinued in most countries where the disease is endemic) [2, 6, 7]. The high toxicity of these drugs and the low rates of parasitological cure once the parasite has spread through multiple organs, and tissues in the vertebrate host are the reason that more effective therapeutic strategies are needed [6, 8]. A potential candidate, preliminarily investigated by our research group, is Suramin (Sur), a urea-derived symmetrical polysulfone with potent antagonist effect on purinergic receptors that is used in the treatment of African human trypanosomiasis (sleeping sickness) [8–10].

From studies *in vitro*, our research group confirmed that Sur acts as a potent inhibitor of *T. cruzi* ecto-nucleoside triphosphate diphosphohydrolase (*ecto*-NTPDase), a member of the CD39 family described in trypanosomatids [10]. This enzyme is essential for the metabolism and survival of *T. cruzi*, also acting as an important factor of parasite virulence [10, 11]. By hydrolyzing extracellular nucleotides (e.g., triphosphates and diphosphates) in infected hosts, *ecto*-NTPDase ensures the salvage of purine rings, which are essential for *T. cruzi* metabolism but are not synthesized by the parasite itself [11]. When investigating Sur as a potential chemotherapy candidate for *T. cruzi* in a preliminary study, we identified that mice infected by *ecto*-NTPDase-inhibited trypomastigotes developed lower parasitemia and higher host survival than animals infected with control parasites [10]. However, when Sur and Bz were combined, mice infected with a virulent strain of *T. cruzi* exhibited increasing parasitemia and myocardial damage [8]. Considering that only the parasites were exposed to Sur in the study by Santos et al. [10], it is possible that Sur has induced a negative effect when administered to infected hosts, modifying the parasitological outcomes [8].

As well as inhibiting *ecto*-NTPDase in *T. cruzi*, Sur also acts as a P2-purinoceptor antagonist in mammalian cells [8, 12]. Purinergic signaling pathways are directly involved in the control of host cell functions, including immune response (e.g., neutrophil and macrophage chemotaxis, recognition of damaged cells, T cell activation, cytokines release, and phagocytosis) [13, 14]. Considering the impact of Sur on parasite and host cell biology, it is surprising that the chemotherapeutic effects and biological mechanisms triggered by Sur in *T. cruzi*-infected hosts remain poorly understood. Therefore, this study was designed to investigate the impact of Sur-based chemotherapy in a murine model of Chagas disease. In addition to parasitism of the blood and heart, the participation of inflammatory mediators, antioxidant defenses, and reactive tissue damage in the pathogenesis of acute Chagas cardiomyopathy was also analyzed.

## 2. Materials and Methods

### 2.1. Animal Model

Ten-week-old male C57BL/6 mice, weighing 26.2 ± 4.1 g were maintained in an animal facility with controlled temperature (22 ± 2°C), relative humidity (60–70%), and light/dark cycles (12/12 h). Food and water were provided *ad libitum*. The Institutional Ethics Committee for Animal Use approved this study (protocol 77/2012). All experimental protocols were conducted according to the guidelines issued by the Brazilian College of Animal Experimentation.

### 2.2. Infection and Treatments

Seventy animals were equally randomized into five groups: SAL = uninfected and untreated; INF = infected and untreated; SR5, SR10, and SR20 = infected and treated with 5, 10, or 20 mg/kg of the purinergic antagonist suramin. The infection was induced by intraperitoneal inoculation of 5000 *T. cruzi* trypomastigotes (Y strain). The parasites were obtained from mice previously infected with metacyclic trypomastigotes obtained from late stationary-phase cultures on liver infusion tryptose medium [8]. The doses of suramin were based on (i) one-fourth, (ii) half, and (iii) the therapeutic dose (Sur, 20 mg/kg/day) for African trypanosomiasis [8, 15]. Suramin was dissolved in sterilized water and intraperitoneally administered for 15 consecutive days after confirmation of the infection by microscopic identification of parasites in blood samples from all inoculated mice. Control mice were concurrently treated with sterilized water. The animals were euthanized 48 h after the last treatment by cardiac puncture after deep anesthesia (ketamine 45 mg/kg and xylazine 5 mg/kg, i.p.).

### 2.3. Parasitemia and Mortality

After inoculation, parasitemia was determined daily by microscopic examination of blood samples (5 *μ*L) obtained from mice tails according to the standardized protocol [16]. The prepatent period (days until the first occurrence of circulating trypomastigotes), mean parasitemia, and the peak of parasitemia was determined. Mortality was recorded as the number and percentage of accumulated deaths within the experimental period [8].

### 2.4. Cardiac Parasite Load

Parasite load was analyzed by quantitative PCR (qPCR) [8]. Briefly, genomic DNA was extracted from heart samples of control mice and infected mice using a DNA purification kit (Assistant®, Promega, USA). Genomic DNA was quantified by spectrophotometry and adjusted to 25 ng/*μ*L. The PCR reaction was adjusted to 10 *μ*L, containing 5 *μ*L of SYBR® Green (Applied Biosystems, Carlsbad, CA, USA), 50 ng of genomic DNA, 0.35 *μ*M of *T. cruzi* repeat DNA-specific primers, or 0.50 *μ*M of tumor necrosis factor-*α* primers (TNF-*α*). Based on Cummings and Tarleton [17], the trimers for murine TNF-*α* were TNR-5411 5′-CAGCAAGCATCTATGCACTTAGACCCC-3′ and TNF-5241 5′-TCCCTCTCATCAGTTCTATGGCCCA-3′; and the primers for *T. cruzi* repetitive DNA were TCZ-R 5′-CCAAGCAGCGGATAGTTCAGG-3′ and TCZ-F 5′-GCTCTTGCCCACAMGGGTGC-3′. The cycling program was adjusted according to Santos et al. [8], and the reactions were performed in a 96-well plate using a standard curve and two negative controls (with DNA from noninfected mice and without DNA). *T. cruzi* DNA levels were normalized as follows: normalized value = (mean DNA *T. cruzi*/mean DNA TNF-*α*) × 1000, where “1000” represents the expected value for TNF-*α* from 30 mg of heart samples. The efficiencies of amplification ([E] = 10^[−1/slope]^) were determined by StepOne™ Software v2.0 [18].

### 2.5. Parasitism of Cardiomyocytes and Cardiac Damage

Heart samples were fixed in 10% formaldehyde (0.1 M, pH 7.2) for 48 h and embedded in histological resin [19]. Blocks were cut into 3 *μ*m thick histological sections and stained with toluidine blue and basic fuchsine. To avoid analyzing similar areas of the heart, the sections were collected in semi-series, using one out of every 50 cuts. For each animal, eight sections were obtained and 24 histological fields were randomly sampled at ×400 and ×1000 magnification, and a total of 10.16 × 10^6^ *μ*m^2^ and 4.26 × 10^4^ *μ*m^2^ cardiac area were analyzed, respectively. Images were captured by bright field photomicroscope (Axio Scope A1, Carl Zeiss, Germany) [20].

Using the stereological method cardiomyocyte parasitism was estimated as the number density (Q) of *T. cruzi* amastigote nests (AN) at ×400 magnification, according to the formula Q_AN_ = ΣAN/At, where At is the dimension of the test area (42.3 × 10^4^ *μ*m^2^). Heart pathological remodeling was estimated as the volume density (Vv (%)) of the main cardiac compartments (parenchyma (PCh) and stroma (SM)). Volume density was measured according to the formula Vv_PCh_ or _SM_, % = Pp/Pt, where Pp is the number of points hitting the compartment of interest and Pt is the total number of points in the test system (*n* = 100). The inflammatory infiltrate was evaluated at ×1000 magnification. The number density of mononuclear (MN) and polymorphonuclear cells (PMN) cells was estimated as Q_MN or PMN_ = ΣMN or ΣPMN/At, where At = 1.78 × 10^3^ *μ*m^2^ [21, 22]. All microstructural analysis was performed using the software Image Pro Plus 4.5.1 (Media Cybernetics, Silver Spring, MD, USA) [22].

### 2.6. Cytokine Immunoassay

Heart samples were homogenized in the presence of a protease inhibitor cocktail (Sigma-Aldrich, St. Louis, MO, USA) and centrifuged for 10 min at 3000×*g*. The supernatant was collected and analyzed in a FACSVerse flow cytometer using a mouse-specific cytometric bead array kit (BD Biosciences, San Diego, USA). The cytokines interleukin-10 (IL-10), interferon-*γ* (IFN-*γ*) and tumor necrosis factor-*α* (TNF-*α*) were measured. Standard curves were obtained for all cytokines from a range of 20–5000 pg/mL. According to the manufacturer and the analyte, the lower limit of cytokine detection was 2.5–52.7 pg/mL [10].

### 2.7. Nitric Oxide Assay

Cardiac levels of nitrite/nitrate (NO_2_^−^/NO_3_^−^) were quantified as an indirect nitric oxide (NO) measure. Using the Griess reaction [23], 50 *μ*L of supernatant from the heart homogenate was added to 50 *μ*L of Griess reagent (2.5% H_3_PO_4_, 0.1% naphthalene diamine dihydrochloride, and 1% sulfanilamide). After 10 minutes of incubation at room temperature, the reaction was analyzed in a microplate spectrophotometer at 550 nm (BioTek Instruments, Inc., Winooski, VT, USA).

### 2.8. Protein, Lipid, and DNA Oxidation

Protein oxidation was estimated by quantifying protein carbonyl in cardiac tissue [24]. Briefly, 0.5 mL of 10 mM dinitrophenylhydrazine (DNPH) was added to tissue pellets. The reaction involved derivatization of the carbonyl group with 2,4-dinitrophenylhydrazine (DNPH), producing a stable 2,4-dinitrophenyl (DNP) hydrazone product. The optical density was then measured in the spectrophotometer at 370 nm.

Malondialdehyde (MDA) was quantified and used as a biomarker of lipid oxidation in cardiac tissue. Heart samples were homogenized in phosphate buffer by being centrifuged for 10 min at 10000×*g.* The homogenate was incubated with a thiobarbituric acid solution (0.25 N HCl, thiobarbituric acid 0.38%, and trichloroacetic acid 15%) for 15 minutes. The formation of MDA was monitored in the spectrophotometer at 535 nm as described by [25].

Tissue levels of 8-hydroxy-2′-deoxyguanosine (8-OHdG) were measured and used as a marker of DNA oxidation [26]. Briefly, heart fragments were incubated at 55°C with 10 mg/mL of proteinase K, and then DNA was extracted by the phenol-chloroform (1 : 1) method [27]. DNA was resuspended in 0.1 mmol/L EDTA and 10 mmol/L Tris-HCl. Sodium acetate buffer (200 mmol/L) and 5 *μ*g nuclease P1 (Aldrich Chemical Co., Milwaukee, USA) were added to 45 *μ*L DNA samples. The reaction was incubated for 1 h at 37°C to digest the molecular chain, producing nucleotides. Then, 0.6 units of alkaline phosphatase, 5 *μ*L of 10 mmol/L MgCl_2_, and 500 mmol/L Tris-HCl were added to hydrolyze the nucleotides into nucleosides. The nucleosides were used for 8-OHdG quantification by enzyme-linked immunosorbent assay—ELISA (Cell Biolabs Inc., San Diego, CA, USA).

### 2.9. Enzymatic and Nonenzymatic Antioxidant Defenses

The activity of antioxidant enzymes glutathione-S-transferase (GST), catalase (CAT), and superoxide dismutase (SOD) was analyzed in heart samples homogenized in ice-cold phosphate buffer (pH = 7.0) by being centrifuged for 15 min at 5°C and 3500×g. The kinetic method of hydrogen peroxide (H_2_O_2_) decomposition was used to evaluate CAT activity [28]. The xanthine oxidase method, which is based on the reduction of nitroblue tetrazolium and the production of H_2_O_2_, was used to estimate SOD activity [29]. GST activity was estimated from the rate of NADPH oxidation, which was analyzed in the spectrophotometer at 340 nm [30]. All results were normalized by protein levels, which were measured in the supernatant using the Bradford method [31].

The nonenzymatic defenses in the heart homogenate were analyzed by using a total antioxidant capacity assay kit, according to the manufacturer's instructions (TAC Assay Kit, Sigma Aldrich, Milwaukee, USA). The method was based on the inhibition of antioxidant enzymes and the prevention of Cu^2+^ oxidation by small antioxidant molecules, which is analyzed in a spectrophotometer at 570 nm. The antioxidant capacity was estimated from a standard curve, using trolox as the antioxidant reference.

## 3. Results

Infected animals and those in the SR5 group presented similar mean parasitemia, peak parasitemia, and mortality rates (*p* > 0.05). These parameters were increased in SR10 and SR20 animals compared to the INF group (*p* < 0.05). Parasitemia and mortality also increased in the SR20 group compared to SR5 animals (*p* < 0.05), Table 1.

**Figure 1:**
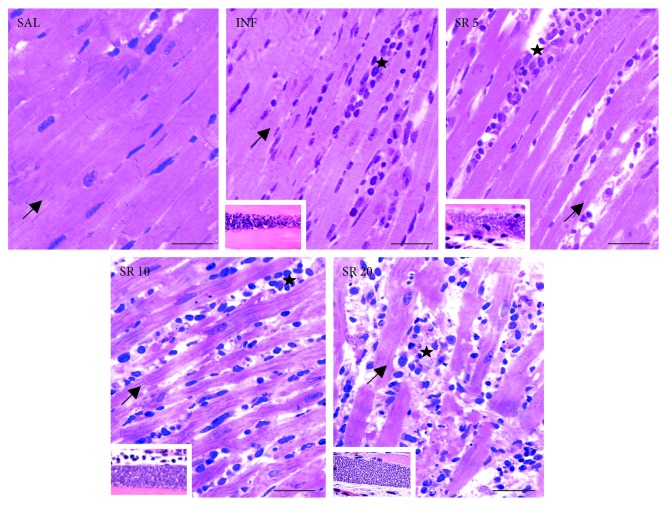
Representative microscopic images of the heart from mice infected with *Trypanosoma cruzi* and treated with the nonselective purinergic antagonist suramin (bright field microscopy, toluidine blue and basic fuchsine staining, bar = 50 *μ*m). In the groups INF, SR5, 10, and 20, the highlighted images represent amastigote nests of *T. cruzi*. Arrows: cardiomyocytes (heart parenchyma), stars: connective tissue (heart stroma) with leucocytes infiltrates. SAL: 0.9% NaCl solution; INF: untreated and infected; SR5, SR10, and SR20: infected and treated with 5, 10, or 20 mg suramin/kg, respectively.

Control animals (SAL) exhibited an organized myocardial structure with parallel cardiomyocytes, scarce connective tissue distribution as interstitial cellularity. All infected animals, especially those in the groups SR10 and SR20, presented amastigote nests in cardiomyocytes, connective tissue expansion, and evident diffuse inflammatory infiltrate (Figure 1).

**Figure 2:**
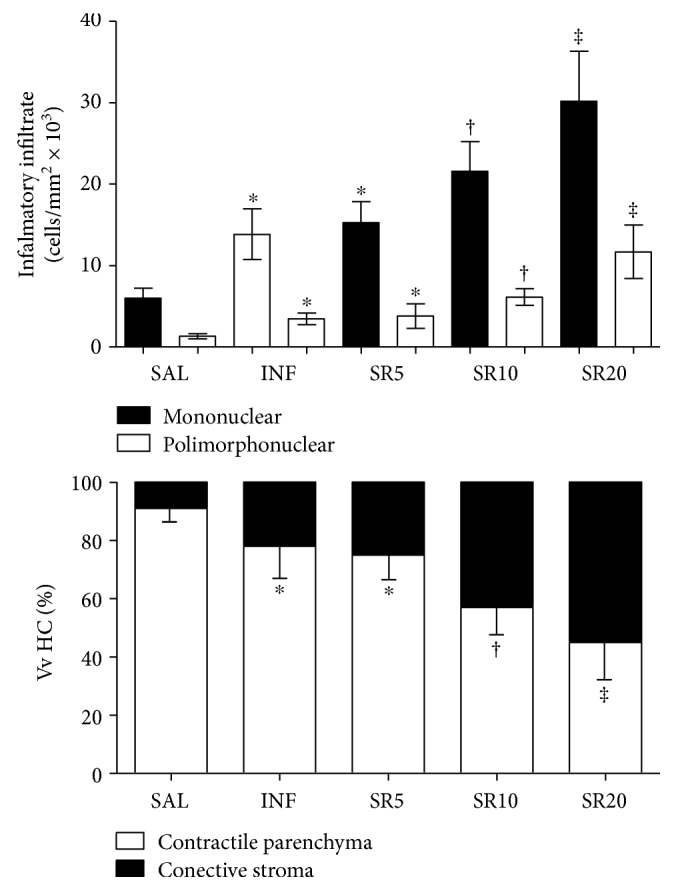
Inflammatory infiltrate and pathological remodeling of the cardiac tissue from mice infected with *Trypanosoma cruzi* and treated with the nonselective purinergic antagonist suramin. Vv Hc (%): volume density of myocardial components. SAL: 0.9% NaCl solution; INF: untreated and infected; SR5, SR10, and SR20: infected and treated with 5, 10, or 20 mg suramin/kg, respectively. ^∗^ † ‡ Statistical difference (*p* < 0.05): compared to ^∗^ SAL; † SAL, INF, and SR5; ‡ SAL, INF, SR5, and SR10.

Cardiac tissue from INF, SR5, and SR10 animals presented an increased distribution of connective stroma, increased mononuclear and polymorphonuclear cell numbers, and a reduced proportion of contractive parenchyma compared to the SAL group. These changes were even more pronounced in SR20 animals compared to the other groups (*p* < 0.05), Figure 2.

**Figure 3:**
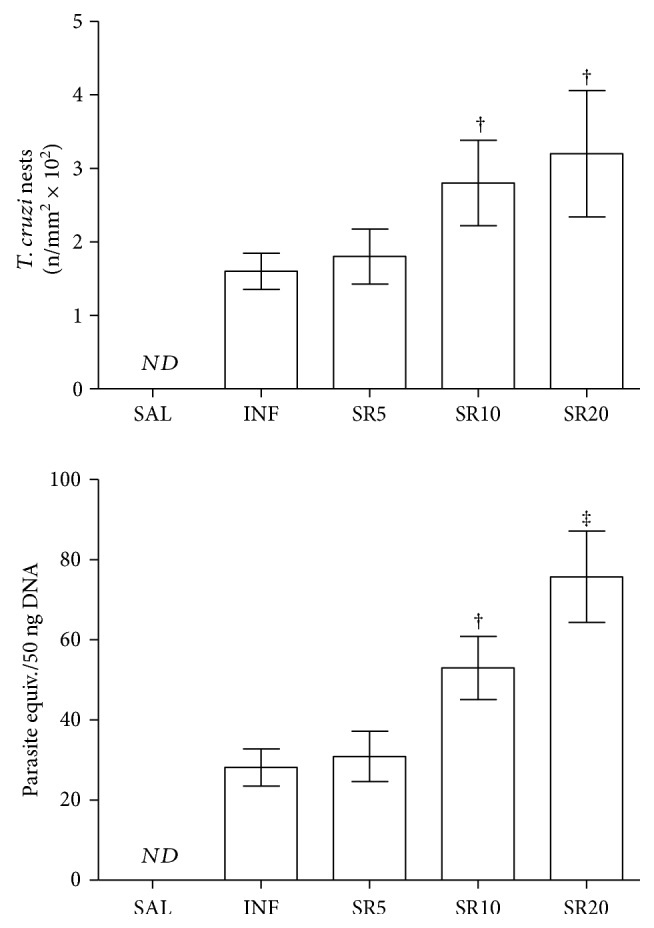
Parasitism, inflammatory infiltrate, and pathological remodeling of the cardiac tissue from mice infected by *Trypanosoma cruzi* and treated with the nonselective purinergic antagonist suramin. Vv Hc (%): volume density of myocardial components. SAL: 0.9% NaCl solution; INF: untreated and infected; SR5, SR10, and SR20: infected and treated with 5, 10, or 20 mg suramin/kg, respectively. † ‡ Statistical difference (*p* < 0.05): compared to † SAL, INF, and SR5; ‡ SAL, INF, SR5, and SR10.

The number of *T. cruzi* nests observed in cardiomyocytes was similar in the groups INF and SR5 but higher in SR10 and SR20 animals (*p* < 0.05). In addition to the observation of amastigote nests through the microscope, the infection was confirmed by PCR in all animals inoculated. The parasite load was similar in INF and SR5 animals but increased in SR10 (*p* < 0.05) and especially in SR20 animals, which exhibited the highest values compared to the other groups (*p* < 0.05), Figure 3.

**Figure 4:**
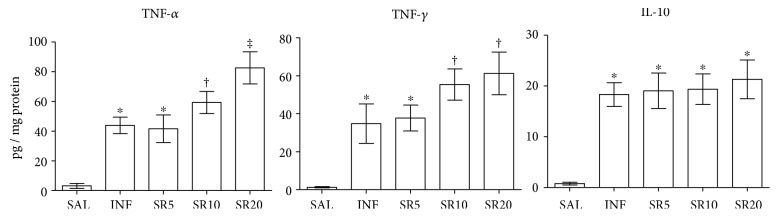
Cytokine cardiac levels in mice infected with *Trypanosoma cruzi* and treated with the nonselective purinergic antagonist suramin. SAL: 0.9% NaCl solution; INF: untreated and infected; SR5, SR10, and SR20: infected and treated with 5, 10, or 20 mg suramin/kg, respectively. ^∗^ † ‡ Statistical difference (*p* < 0.05): compared to ^∗^ SAL; † SAL, INF, and SR5; ‡ SAL, INF, SR5, and SR10.

TNF-*α*, IFN-*γ*, and IL-10 levels were increased in all infected groups compared to SAL. TNF-*α* was similar in the groups INF and SR5 and increased in SR10 (*p* < 0.05) and especially in SR20 animals, which exhibited the highest values compared to the other groups (*p* < 0.05). IFN-*γ* was similar in the groups SR10 and SR20 but higher than INF and SR5 animals (*p* < 0.05). IL-10 levels were similar in all infected animals (*p* < 0.05), Figure 4.

**Figure 5:**
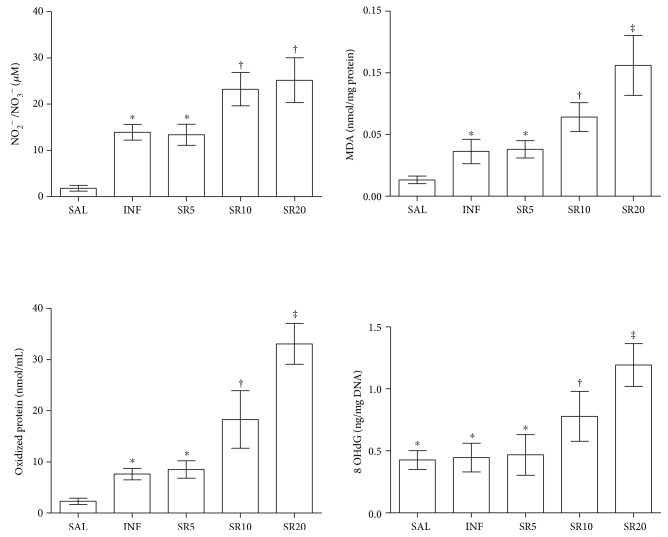
Cardiac levels of nitric oxide (NO), lipid, protein, and DNA markers of oxidative tissue damage in mice infected with *Trypanosoma cruzi* and treated with the nonselective purinergic antagonist suramin. NO was estimated as nitrite/nitrate (NO_2_^−^/NO_3_^−^) levels. MDA, malondialdehyde. Oxidized proteins were estimated as protein carbonyl levels. 8-OHdG, 8-hydroxy-2′-deoxyguanosine. SAL: 0.9% NaCl solution; INF: untreated and infected; SR5, SR10, and SR20: infected and treated with 5, 10, or 20 mg suramin/kg, respectively.

Nitric oxide, MDA, and PCn levels were increased in all infected groups compared to SAL (*p* < 0.05). MDA and PCn levels were also increased in SR10 compared to SR5 and in SR20 compared to all groups (*p* < 0.05). Nitric oxide was similarly high in SR10 and SR20 animals (*p* > 0.05) but higher than the other groups (*p* < 0.05). 8-OHdG levels were similar in the groups SAL, INF, and SR5 but increased in SR10 (*p* < 0.05) and especially in SR20 (*p* < 0.05), Figure 5.

**Figure 6:**
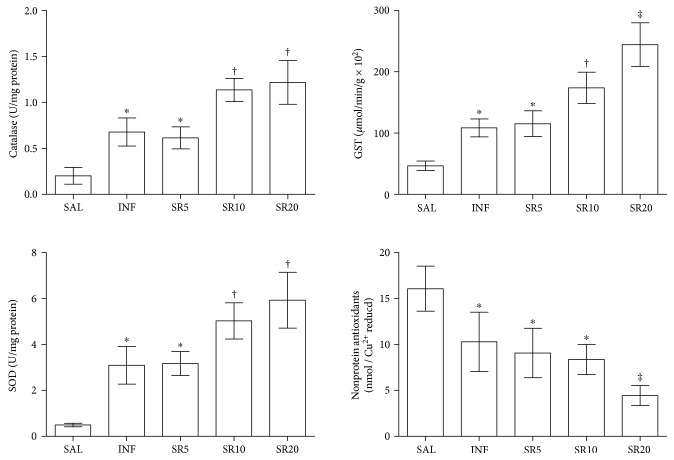
Enzymatic and nonenzymatic antioxidant defenses in mice infected with *Trypanosoma cruzi* and treated with the nonselective purinergic antagonist suramin. GST: Glutathione-S-transferase; SOD: superoxide dismutase. SAL: 0.9% NaCl solution; INF: untreated and infected; SR5, SR10, and SR20: infected and treated with 5, 10, or 20 mg suramin/kg, respectively.

The activity of the enzymes CAT, SOD, and GST was increased in all infected animals when compared to SAL (*p* < 0.05). While enzymatic activity was similar in the INF and SR5 groups, SR10 and SR20 animals exhibited higher values compared to the other groups (*p* < 0.05). The antioxidant activity of nonprotein molecules was reduced in all infected animals compared to the SAL group (*p* < 0.05), and especially in the group S20, which had the lowest values compared to the other groups (*p* < 0.05), Figure 6.

## 4. Discussion

Although Sur has been considered to be a potential strategy to modify *T. cruzi* biology, attenuating host cell infectivity and death *in vitro* [32, 33], when administered to infected murine hosts, this drug produced poor outcomes. Our understanding of the mechanisms associated with the divergent findings reported by *in vitro* and *in vivo* studies is still limited. Currently, the evidence that supports the antitrypanosomal effect of Sur is almost exclusively based on *in vitro* studies [34, 35]. For example, Bisaggio et al. [33, 36] showed that the antitrypanosomal effect induced by Sur treatment *in vitro* is mediated by direct *T. cruzi* morpho-functional damage (e.g., slow kinetoplast migration and cell division, reduced motility, flagellar degeneration, and detachment form the cell body), limiting the infection of LLC-MK2 host cells. Santos et al. [10] also reported that *T. cruzi* trypomastigotes pretreated with Sur presented reduced infective potential in Vero cells, which was closely correlated with marked parasite ecto-NTPDase1 inhibition. As Sur-mediated Mg^2+^-dependent *ecto*-ATPase inhibition markedly reduced cell adhesion and internalization of epimastigotes and trypomastigotes forms by isolated murine macrophages, this enzyme has been considered an important virulence factor in *T. cruzi* [35].

From *in vitro* evidence, it is understandable that *ecto*-NTPDase has been considered a promising molecular target in the treatment of Chagas disease [10, 37, 38]. However, *in vitro* studies also showed a contradictory response. While low doses of Sur were associated with effective enzymatic inhibition and reduced infectivity on murine peritoneal macrophages, surprisingly, high doses induced exacerbated enzymatic activity and intense macrophage infection by *T. cruzi* [35]. It is still unclear if this increased infectivity is an isolated response to the upregulation of *ecto*-NTPDase activity by *T. cruzi* in an effort to overcome the primary Sur-induced enzymatic inhibition, or if it is due to the blockage of purinergic signaling pathways in host cells, which also regulates the antimicrobial activity of leukocytes [35, 39, 40]. Regardless of whether the cause is parasite compensation in response to Sur inhibition or blockage of purinergic signaling pathways in host cells, these effects alone or combined can be dangerous to the host. In both cases, *T. cruzi* infection may be amplified, a phenomenon potentially related to the poor experimental outcomes observed in our *in vivo* model. Considering our parasitological findings (parasitemia, *T. cruzi* nests, and parasite load) and mortality rates, there is no doubt that *T. cruzi* was exposed to a more favorable microenvironment in Sur-treated animals. Especially in those groups treated with the highest doses of Sur (10 and 20 mg/kg), higher parasitemia and mortality were associated with intense cardiac parasitism, marked myocarditis with diffuse inflammatory infiltrate, reduced distribution of the cardiac contractile parenchyma, and compensatory expansion of the connective stroma.

In the present study, beyond the increase in the number of amastigote nests, Sur treatment amplified the parasite load in cardiac tissue. Interestingly, the results regarding parasite load were correlated with the severity of heart inflammation and oxidative stress, which were potentially related to the dose-dependent microstructural changes induced by Sur. The quantification of *T. cruzi* DNA using PCR has been consistently used to evaluate the cardiac parasite load [8, 41]. In addition to its diagnostic value, parasite DNA load is a good predictor of leukocyte infiltration and damage to heart microstructure [41, 42]. Consistent with the parasite load and leucocytes infiltrate, our results also indicated that animals treated with the highest doses of Sur exhibited high cardiac levels of TNF-*α* and IFN-*γ*, which are two effector molecules produced from the polarized Th1 immunological phenotype [43, 44]. In addition to the direct leucocyte recruitment triggered by cytokines (e.g., TNF-*α*, IFN-*γ*) and chemokines (e.g., MCP1, MIP-1 and 2) secreted in response to *T. cruzi* antigens, direct cardiomyocytolysis, and cell death induced by the continuous replication of amastigotes also contributes to leucocyte recruitment and activation [45, 46]. There is evidence that high parasite load is closely correlated with intense immunological polarization to a Th1 phenotype, which is paradoxically implicated in antitrypanosomal defense and cardiac damage [19, 26, 44]. So, although cell-mediated immunity and the Th1-phenotype are the main line of defense against *T. cruzi* [43, 44], exacerbated leucocyte infiltration and the overproduction and extravasation of lysosomal hydrolases and reactive species (e.g., NO, H_2_O_2_, HClO, O_2_^·−^, OH^·−^, and ONOO^·−^) from the “respiratory burst” result in intense lipid, protein, and DNA damage in host cells [47, 48]. Due to parasite persistence and continuous immunological activation, oxidative stress remains present throughout infection, generating more reactive molecular damage and sustained proinflammatory stimuli in a self-sustaining process [7, 8]. Therefore, as the intensity of the immunological response and oxidative stress are related to cumulative reactive tissue damage [8, 26], it is not surprising that the intense myocarditis observed in Sur-treated animals is associated with higher mortality rates.

It has been proposed that the increased extracellular release of ATP (eATP) [37, 49] from the lysis of *T. cruzi*-infected cells could trigger the secretion of proinflammatory cytokines and the inflammatory response from the activation of type 2 purinergic receptors (P2) in resident leucocytes [8, 32]. In fact, this process is highly relevant in the process of death, since high eATP levels act as a molecular signal to activate immune cells, especially macrophages [50]. Considering that eATP cleavage by *T. cruzi* ectonucleotidases attenuates immunological activation, we would expect that parasite *ecto*-NTPDase inhibition by Sur could amplify the immunological response [8]. Although ATP levels have not been evaluated, our findings of TNF-*α*, IFN-*γ*, and leucocyte myocardial infiltration in animals treated with Sur (especially at 10 and 20 mg/kg) were consistent with this proposition. It is tempting to believe that Sur-induced immune hyperactivation may increase host resistance to infection. However, a contrary effect was observed in our model. Thus, once the exacerbated immune response is linked to redox imbalance and intense cardiac reactive stress [47, 48], by potentiating myocarditis, Sur can reinforce cardiac damage and mortality rates in infected mice.

As for inflammatory pathology, our results for cytokines and leucocyte infiltrate were supported by Zacks et al. [51] and Gupta et al. [52]. These authors showed severe myocarditis in *T. cruzi-*infected mice, with intense IL-1*β* and TNF-*α* production by macrophages and IFN-*γ* production by CD4^+^ and CD8^+^ T cells. It is a general characteristic of inflammatory processes that proinflammatory and anti-inflammatory molecules are produced simultaneously in order to achieve an adequate balance among Th1, Th2, and Treg responses, which is a balance that is specific in different infectious diseases [43, 53]. IL-10 is an important anti-inflammatory cytokine, which was upregulated in all infected and treated animals. Although this molecule exerts a central regulatory function in attenuates Th-1 phenotype in *T. cruzi* infection [43, 53, 54], its increased levels were not enough to counteract heart damage in our model. To date, the differential regulation that Sur exerts on the cytokines produced in Chagas disease is poorly understood, which is an issue that requires further investigation.

Besides discovering a proinflammatory profile, we also identified that Sur was favorable to NO production (an additional and important Th1 effector) and lipid (MDA), protein (PCn), and DNA (8-OHdG) oxidation in *T. cruzi*-infected mice. There is consistent evidence that oxidative and nitrosative stress is intensely upregulated in *T. cruzi* infection, producing a marked redox imbalance and a cytotoxic cardiac microenvironment [43, 47, 52, 55]. In addition to recruited leucocytes, cardiomyocytes are also a primary source and target of reactive mediators, whose production is exacerbated due to enzymatic decoupling in the electron transport chain of degenerated mitochondria in infected cells [52]. Unsaturated fatty acids in the plasma membrane and cell organelles are direct targets of reactive species, leading to the production of lipid peroxyl radicals (LOO^·−^), alkoxy radicals (RO^·−^), and MDA, which propagate oxidative damage in a process called lipid peroxidation [56, 57]. Together with MDA, protein carbonyl groups are useful biomarkers of oxidative stress, especially because of the early formation and stability of these molecules [58]. In Chagas disease, these molecules are classical markers of regional and systemic indicators of oxidative tissue damage, whose levels are potentially modulated by antioxidant and anti-inflammatory agents [7, 26]. However, protein and lipid oxidation are not always associated with increased 8-OHdG levels, since DNA damage usually occurs in more intense oxidative processes, which outweigh the mechanisms of DNA repair [26]. Considering that proinflammatory cytokines potentiate the production of reactive mediators in leucocytes and cardiomyocytes [59–61], the increased TNF-*α* and IFN-*γ* heart levels in animals treated with Sur was consistent with pronounced reactive molecular damage, which exhibits a strong correlation with the degree of severity of myocarditis [55, 62].

As observed in the present study, increased activity of antioxidant enzymes is expected in acute *T. cruzi* infections [46, 52, 55]. There is evidence that this response occurs as an adaptive mechanism of the heart in an attempt to counteract reactive tissue damage and the death of cardiomyocytes [8, 26]. The coupled increase in oxidative damage and activity of antioxidant enzymes was not surprising since the activation of cell signaling pathways by tissue levels of oxygen (ROS) and nitrogen (RNS) reactive species positively modulates gene expression and the activity of antioxidant enzymes [63]. However, this compensatory response was not effective in blocking cardiac molecular damage in infected animals, especially the groups treated with the highest doses of Sur (10 and 20 mg/kg). There is no doubt that CAT, SOD, and GST are central antioxidant enzymes directly involved in protecting the heart against *T. cruzi*-induced reactive tissue damage [7, 26]. SOD is the first line of defense against ROS, catalyzing the dismutation of O_2_^·−^ into O_2_ and H_2_O_2_. In combination with CAT, H_2_O_2_ is degraded into H_2_O and O_2_. In addition, GST is also a notorious radical scavenger, catalyzing the conjugation of reactive species with glutathione [64–66]. Unlike acute infections, chronic Chagas disease is often associated with the downregulation of antioxidant enzymes. This process seems to be related to the enzymatic exhaustion created by sustained oxidative stress, which is closely related to the severity of chronic Chagas cardiomyopathy [26, 67].

Curiously, levels of nonprotein antioxidants in the heart were reduced in all infected animals with drastic depletion in animals treated with Sur at 20 mg/kg. Nonprotein antioxidants are mainly low molecular weight substances (e.g., C and E vitamins, carotenoids, and uric acid) with important roles in redox balance [68–70]. Unlike endogenous antioxidant enzymes, nonprotein antioxidants are not a direct genic product. Thus, instead of positive modulation in response to reactive tissue damage, *T. cruzi* infection and severe myocarditis are associated with the depletion of nonprotein antioxidants in animals [26, 68, 69] and humans [70, 71]. However, the question of whether the exogenous administration of nonprotein antioxidants such as vitamins C and E can be beneficial in attenuating Chagas cardiomyopathy remains controversial [69–71]. In a recent study by our research group, although vitamin supplementation restored the heart levels of vitamin C and E, the treatment was ineffective in attenuating cardiac inflammation and oxidative stress [26]. Similar results were reported by de Gusmão et al. [68] and Marim et al. [69], reinforcing our proposition that controlling inflammation, the main source of reactive species, is a more rational strategy to attenuate oxidative stress and heart damage than a direct antioxidant approach [26]. Thus, it seems reasonable to assume that depletion of nonprotein antioxidants in Sur-treated animals creates favorable conditions for reactive molecular damage, contributing to cardiomyocytes death, myocarditis development, and increased general mortality.

Taken together, our findings indicated that despite the potent antiparasitic effects reported from *in vitro* models, Sur-based chemotherapy delivers poor outcomes in mice infected by *T. cruzi*. To date, there is limited understanding of whether, and to what extent, the observed effects are derived from a negative impact of Sur on the purinergic signaling pathways of host cells. However, our findings clearly demonstrate that by enhancing parasitism, inflammation, and reactive tissue damage, Sur-based chemotherapy aggravates myocarditis and increases mortality in *T. cruzi*-infected mice, contradicting the relevance *in vitro* theoretically attributed to this drug for the treatment of *in vivo* models of Chagas disease. These findings indicate that although Sur is relevant for the treatment of African trypanosomiasis, its applicability to the treatment of American trypanosomiasis is questionable and should be analyzed with caution.

## Figures and Tables

**Table 1:** Parasitological parameters and mortality in mice infected with *T. cruzi* and treated with different doses of the nonselective purinergic antagonist suramin.

Groups	Prepatent period (days)	MP (parasites ×10^3^/0.1 mL blood)	PP (parasites ×10^3^/0.1 mL blood)	Mortality (*n*/%)
SAL	*ND*	*ND*	*ND*	0/0%
INF	5	27.11 ± 15.08	49.17 ± 20.15	5/35.7%
SR5	5	29.16 ± 11.31	53.55 ± 20.37	5/35.7%
SR10	4	45.71 ± 10.94^∗^	80.13 ± 48.52^∗^	7/50%
SR20	4	58.83 ± 15.40^∗^†	117.25 ± 60.0^∗^†	9/64.3%

Data are reported as mean ± standard deviation. MP, mean parasitemia; ND, not detected; PP, peak of parasitemia; SAL, control noninfected (0.9% NaCl solution); INF, untreated infected; SR5, 10, and 20; groups treated with suramin at 5, 10, or 20 mg/kg, respectively. ^∗^†Statistical difference (*p* < 0.05), ^∗^ compared to INF and † compared to INF and SR5; Kruskal-Wallis test.

## Data Availability

All data used to construct the manuscript are presented in the submitted file.
